# Genome-Scale Nuclear Markers Provide Strong Evidence for Species-Level Differentiation between the Mahseer Fishes *Tor tambra* and *Tor tambroides*

**DOI:** 10.34133/csbj.0112

**Published:** 2026-05-28

**Authors:** Komwit Surachat, Krittaporn Lempan, Chanida Sakunrang, Monwadee Wonglapsuwan

**Affiliations:** ^1^ Department of Biomedical Sciences and Biomedical Engineering, Faculty of Medicine, Prince of Songkla University, Hat Yai, Songkhla 90110, Thailand.; ^2^ Division of Biological Science, Faculty of Science, Prince of Songkla University, Hat Yai, Songkhla 90110, Thailand.; ^3^ Center for Genomics and Bioinformatics Research, Faculty of Science, Prince of Songkla University, Hat Yai, Songkhla 90110, Thailand.

## Abstract

Accurate discrimination between closely related mahseer species remains a persistent taxonomic challenge, particularly for *Tor tambra* and *Tor tambroides*, which exhibit strong morphological similarity and limited resolution using mitochondrial DNA markers. In this study, we generated high-quality hybrid genome assemblies for male and female *T. tambra* and performed genome-wide comparative analyses to identify nuclear loci capable of distinguishing these species. Despite high overall genomic similarity and strong reciprocal mapping rates, a mapping-based unmapped-read discovery framework identified candidate nuclear regions exhibiting consistent asymmetric read support between taxa. These analyses indicate that strongly asymmetric candidate regions are highly localized and, under the applied detection framework, represent only a small fraction of the reference genome. Phylogenomic reconstruction based on conserved Benchmarking Universal Single-Copy Orthologs robustly resolved *T. tambra* and *T. tambroides* as distinct, well-supported nuclear lineages, while Mash-based genome-wide comparisons revealed consistent but shallow divergence (≈1.5%), supporting recent evolutionary separation. Genome-informed primer design and multi-individual validation confirmed one robust species-specific nuclear marker and additional loci containing fixed interspecific nucleotide differences. Furthermore, *k*-mer-based analysis detected a female-biased enrichment signal under stringent filtering conditions, although this observation requires validation across multiple individuals. Collectively, these findings provide robust genome-scale evidence supporting species-level differentiation between *T. tambra* and *T. tambroides*, while demonstrating that divergence is highly localized within an otherwise conserved genomic background. Together with previous mitochondrial evidence showing limited discriminatory power for this species pair, this study establishes a reproducible nuclear genome-based framework for species delimitation and diagnostic marker development in recently diverged teleost lineages.

## Introduction

The genus *Tor* (Cyprinidae), commonly referred to as mahseer, comprises large-bodied freshwater fishes of considerable ecological, economic, and cultural importance across South and Southeast Asia [1]. Several *Tor* species are of conservation concern due to habitat degradation, hydrological modification, and overexploitation. Among them, *Tor tambra* and *Tor tambroides* are particularly important for fisheries, aquaculture development, and regional biodiversity management [1–3]. However, reliable discrimination between these taxa remains problematic because of strong morphological similarity and overlapping meristic and morphometric characters [4]. Molecular studies based on mitochondrial DNA markers have further highlighted low genetic divergence and inconsistent species boundaries, reinforcing the taxonomic complexity within the genus *Tor* [2].

Traditional taxonomic approaches in mahseer, including species of *Tor*, have relied heavily on morphological diagnostics and mitochondrial DNA (mtDNA) markers [5–7]. Although mtDNA-based barcoding (e.g., cytochrome *c* oxidase subunit I [*COX1*] and cytochrome *b*) has proven useful for many teleost lineages [8], single-locus mitochondrial markers often lack sufficient resolution in recently diverged taxa [9]. Incomplete lineage sorting, shared ancestral polymorphism, and mitochondrial introgression can obscure true species boundaries, particularly in groups characterized by shallow divergence times and large effective population sizes [10]. In mahseer, especially within *Tor*, these factors have contributed to persistent uncertainty in species delimitation and phylogenetic interpretation [4,7,11,12].

Genome-wide nuclear data can resolve evolutionary relationships more accurately because they combine information from thousands of independently inherited loci rather than relying on a single marker [13]. This contrasts with mitochondrial genomes, which provide a single maternally inherited haplotype and thus a more limited representation of evolutionary history [14]. Nuclear genomes, by capturing biparental inheritance, offer a broader and more accurate picture of evolutionary relationships [15,16]. The field of phylogenomics leverages these extensive genomic datasets to reconstruct evolutionary trees with greater accuracy, especially in complex cases where traditional methods struggle [17,18].

Advances in long-read sequencing technologies and hybrid assembly strategies now enable the generation of high-quality reference genomes for nonmodel teleost species [19–21]. These developments have facilitated genome-scale comparative analyses, phylogenomics, and systematic marker discovery at unprecedented resolution [15,18–20].

In recently diverged lineages, species boundaries may not be characterized by genome-wide differentiation but rather by localized regions of elevated divergence embedded within otherwise highly similar genomes [22,23]. Such “genomic islands” of differentiation have been documented in multiple teleost systems and may arise through selection, structural variation, or reduced recombination [23]. Therefore, genome-scale approaches that evaluate both global phylogenetic signal and localized sequence asymmetry are particularly valuable for clarifying species delimitation in morphologically conserved and recently radiated groups such as *Tor*.

In this study, we generated hybrid genome assemblies for male and female *T. tambra* and used genome-scale nuclear analyses to investigate differentiation between *T. tambra* and *T. tambroides*. We combined phylogenomic reconstruction, genome-wide similarity analysis, and a mapping-based marker-discovery framework to evaluate whether the 2 taxa can be resolved as distinct nuclear lineages and to identify candidate diagnostic loci. Together, this study provides genome-scale evidence for species-level differentiation and establishes a framework for nuclear marker development in recently diverged teleost lineages.

## Materials and Methods

### Ethical considerations

The specimens involved in this study were managed in accordance with the protocols outlined by the Animal Ethics Committee of Prince of Songkla University, Hat Yai, Songkhla, Thailand (Ref. AQ026/2023).

### Sample collection and DNA extraction

One male and one female *T. tambra* specimen were obtained by wild capture from Chanae District, Narathiwat Province, Thailand (5.986149°N, 101.567395°E). Sex of the specimens was confirmed prior to downstream analysis by gonadal histology following anesthesia, as described in our previous study [24]. High-molecular-weight genomic DNA was extracted from skeletal muscle tissue using the QIAquick Genomic DNA Kit (QIAGEN, USA) according to the manufacturer’s instructions. DNA quality and quantity were assessed prior to downstream library preparation, and purified DNA was stored at −20 °C until further use.

### Library preparation and whole-genome sequencing

For long-read sequencing, 1 μg of high-molecular-weight, unsheared genomic DNA was processed using the LSK109 ligation sequencing kit (Oxford Nanopore Technologies, UK) according to the manufacturer’s protocol. Each specimen’s library underwent sequencing on 2 MinION flow cells. Nanopore reads were base-called from their fast5 files using Guppy version 4.4.1 (in high accuracy mode) to generate fastq files. Additionally, the sequence libraries of short reads generated by a NovaSEQ6000 (Illumina, San Diego, CA) with 2 × 150 bp were obtained from a previous study [24] for hybrid analysis.

### Hybrid genome assembly

The trimmed short-read sequences from our previous study were combined with long-read sequences generated in the present work. Oxford Nanopore Technologies reads were trimmed using Porechop [25] to improve sequence quality and remove adapter contamination. A hybrid de novo genome assembly was performed using MaSuRCA v4.1.2 [26] with one paired-end Illumina library (expected insert size 500 bp; SD 50 bp) and Oxford Nanopore long reads as input. The assembly was run with GRAPH_KMER_SIZE=auto, LHE_COVERAGE=25, LIMIT_JUMP_COVERAGE=300, CA_PARAMETERS=cgwErrorRate=0.15, NUM_THREADS=50, CLOSE_GAPS=1, SOAP_ASSEMBLY=0, and FLYE_ASSEMBLY=0; the full configuration file is provided in Script S1.

To assess the quality of the assembled *T. tambra* genomes, various assembly metrics were evaluated using QUAST v5.0.2 [27], with parameters set to a read length of 150 bp and a maximum *k*-mer coverage of 1,000. Additionally, the completeness of the assembled *T. tambra* genomes and other publicly available fish genomes was assessed using Benchmarking Universal Single-Copy Orthologs (BUSCO) v5.2.2 [28], based on the actinopterygii_odb10 database, which comprises conserved single-copy orthologs from ray-finned fishes.

Prior to gene prediction, a species-specific repeat library was generated de novo using RepeatModeler v2.0.1 [29], and the assembled genome was subsequently soft-masked with RepeatMasker v4.1.0 using the custom repeat library before downstream RNA-sequencing (RNA-seq) alignment and BRAKER2 annotation.

### Gene prediction and functional annotation

To enhance the accuracy of gene prediction in *T. tambra*, transcriptomic data from a closely related species was incorporated into the gene prediction pipeline. Publicly available *T. tambroides* RNA-seq data were retrieved from the National Center for Biotechnology Information (NCBI) Sequence Read Archive (SRA) under accession SRR14520879 from the study of Lau et al. [19]. The raw RNA-seq reads were quality filtered using Trimmomatic v0.33 [30] with default parameters to remove adapter sequences and low-quality bases. The high-quality, filtered reads were then aligned to the repeat-masked draft genome assembly using HiSAT2 [31], a splice-aware aligner optimized for transcriptomic data. The resulting transcriptome alignment files in Binary Alignment/Map format, together with the soft-masked genome assembly, were used as input for BRAKER2 v2.1.6 [32]. BRAKER2 was run with RNA-seq alignment evidence on the soft-masked genome (--softmasking), and gene models were exported in General Feature Format version 3 format (--gff3). BRAKER2 integrates GeneMark-EX and AUGUSTUS, leveraging both ab initio prediction and extrinsic evidence from RNA-seq alignments to improve the accuracy and completeness of protein-coding gene prediction. This approach allowed us to refine gene models by providing evidence-based training for gene prediction algorithms and accurately delineating exon–intron boundaries within the *T. tambroides* genome. The full BRAKER2 command used for structural annotation is provided in Script S2.

Functional annotation of the predicted protein-coding genes was performed using eggNOG-mapper v2 [33], an automated tool for fast functional annotation based on orthology assignment. The protein sequences predicted by BRAKER2 were used as input, and annotation was conducted against the eggNOG v5.0 database, which contains orthologous groups and functional annotations covering a broad range of taxonomic groups, including vertebrates and ray-finned fishes. The analysis was run with default parameters, enabling the assignment of Gene Ontology terms, Clusters of Orthologous Groups (COG) functional categories, Kyoto Encyclopedia of Genes and Genomes (KEGG) pathway annotations, and predictions of associated protein domains. This functional annotation process provided insight into the biological roles, metabolic functions, and cellular pathways associated with the predicted genes in *T. tambra*, facilitating downstream comparative genomics and evolutionary analyses.

To further characterize genome architecture, repetitive elements were annotated using a de novo repeat-identification workflow. A combined custom repeat library was generated from the male and female *T. tambra* primary genome assemblies using RepeatModeler 2.0.7 [29], and repeat-family classification was subsequently performed. The classified custom library was then used in RepeatMasker v4.2.3 [29] to annotate repetitive elements in each assembly. Repeat classes were summarized into major categories, including retroelements, DNA transposons, unclassified interspersed repeats, simple repeats, satellites, and low-complexity regions. Because the analysis was based on a de novo custom library, a proportion of repeat families remained classified as unknown and was interpreted accordingly.

### Exploratory analysis of sex-associated sequence variation by read-depth comparison

To explore candidate sex-associated genomic regions, male and female Illumina paired-end reads were mapped to a common reference assembly (female genome assembly used as reference) and compared using scaffold-level coverage profiles. Prior to mapping, the reference was indexed and reads were aligned using BWA-MEM v0.7.19 [34]. Alignments were coordinate sorted and indexed with SAMtools v1.21 [35]. Genome-wide sequencing depth and coverage uniformity were assessed from alignment summaries and average depth per scaffold was estimated using mosdepth v0.3.3 [36]. For each scaffold, the mean depth was calculated, and male-to-female depth ratios were computed after adding a small pseudocount to stabilize ratios at low coverage. Candidate sex-associated scaffolds were defined as those showing pronounced sex-biased coverage patterns. To reduce false positives driven by short contigs and stochastic mapping variation, analyses were repeated after filtering scaffolds to retain only those ≥50 kb in length, and only these length-filtered candidates were prioritized for downstream interpretation and gene-content inspection.

### Sex-specific *k*-mer analysis from raw reads

*K*-mer-based analysis of sex-associated sequence variation was performed using KMC v3.2.1 [37]. *K*-mer databases were constructed separately for male and female Illumina paired-end reads using a *k*-mer size of 21 (*k* = 21), which provides a balance between sensitivity and specificity for vertebrate genomes. *K*-mer counting was conducted with a minimum occurrence threshold of 3 (-ci3) to reduce the influence of sequencing errors. Computations were performed using multithreading (-t16). Sex-specific *k*-mers were identified by set subtraction between male and female *k*-mer databases, and the resulting *k*-mer sets were used for downstream analysis and mapping to the reference genome.

### Assembly validation by read mapping (supporting quality control)

To validate assemblies and assess read support across scaffolds, male and female Illumina reads were aligned to the chosen reference assembly using BWA-MEM v0.7.19, followed by sorting and indexing with SAMtools v1.21 [35]. Mapping statistics (e.g., overall alignment rate and properly paired reads) were computed to confirm data quality and alignment consistency. Depth distribution and breadth of coverage were summarized using mosdepth [36] v0.3.3 outputs, including global depth distribution and scaffold-level mean depth. These metrics were used to identify scaffolds with low or absent read support and to interpret sex-specific coverage patterns in the context of assembly completeness and potential unplaced/haplotig content.

### Identification of candidate nuclear markers distinguishing *T. tambra* and *T. tambroides* using mapping-based unmapped-read discovery

To identify nuclear genomic regions with diagnostic potential between *T. tambra* and *T. tambroides*, we implemented a mapping-based unmapped-read and coverage-asymmetry discovery strategy leveraging cross-species alignment profiles.

#### Cross-species read mapping and coverage profiling

Paired-end Illumina reads from male and female *T. tambra* individuals were independently aligned to the *T. tambroides* reference assembly using BWA-MEM v0.7.19 [34] under high-stringency parameters. Alignments were coordinate sorted and indexed using SAMtools v1.21 [35]. Per-contig sequencing depth and coverage breadth were quantified using samtools coverage.

Genome-wide weighted mean depth was calculated across all reference contigs to establish a conserved coverage baseline. Contigs exhibiting substantially reduced mean depth relative to the genome-wide mean were identified using a threshold of <30× coverage in pooled mapping data, corresponding to approximately <0.4× of the normalized genome-wide depth. This threshold was selected to capture contigs showing consistent underrepresentation across both male and female individuals while excluding stochastic low-coverage noise.

Contigs meeting the low-coverage criterion were further examined for coverage breadth and consistency between sexes to ensure that reduced depth was reproducible and not sex specific or sample specific.

To assess robustness of the mapping-based estimate, zero-coverage analyses were repeated under 3 alignment-quality thresholds (mapping quality ≥10, ≥20, and ≥30), and summary statistics were compared between male and female *T. tambra* read sets.

#### Extraction of zero-coverage intervals

Within candidate low-coverage contigs, genomic intervals lacking mapped reads were identified using BEDTools v2.30.0 [38] genome coverage profiling. Regions with zero read support were extracted from pooled alignment data to minimize stochastic sampling effects. Only intervals of sufficient length were retained for downstream analysis to avoid inclusion of isolated single-base or short alignment gaps.

Zero-coverage segments were considered candidate-species-associated regions potentially representing sequences absent, highly diverged, or structurally differentiated in *T. tambra* relative to the *T. tambroides* reference genome.

#### Unmapped-read assembly and reciprocal validation

In parallel, read pairs from *T. tambra* that failed to align to the *T. tambroides* reference were extracted. Following quality filtering to remove low-complexity and low-quality reads, unmapped reads were assembled de novo using SPAdes v4.2.0 [39]. Contigs ≥300 bp were retained for downstream screening.

Assembled contigs were subjected to reciprocal alignment against the *T. tambroides* reference to confirm absence or minimal alignment under permissive thresholds. Contigs were additionally evaluated for read-depth support when mapped back to the *T. tambra* assembly to confirm presence and integrity within the source genome.

This dual strategy—(a) low-coverage reference contig screening and (b) de novo assembly of unmapped reads—provided complementary approaches for identifying candidate nuclear loci with strong species-associated signals while minimizing false positives arising from assembly fragmentation, repetitive sequence content, or mapping artifacts.

#### Primer design and experimental validation

**Fig. 1.**
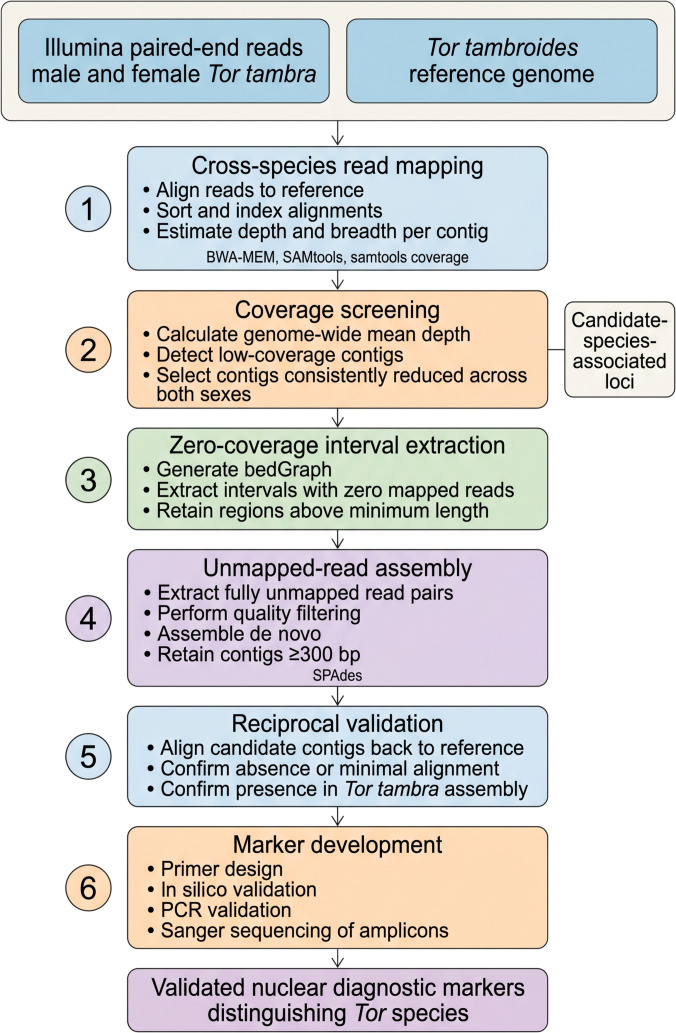
Workflow for mapping-based discovery of candidate nuclear markers distinguishing *T. tambra* and *T. tambroides*. Illumina reads from male and female *T. tambra* were mapped to the *T. tambroides* reference genome to identify low-coverage contigs, zero-coverage intervals, and contigs assembled from unmapped reads. Candidate loci were then reciprocally validated and experimentally tested to identify diagnostic nuclear markers.

Candidate contigs and zero-coverage intervals were prioritized based on length, reproducibility across individuals, and absence of high-confidence matches in the alternative species reference. Primer pairs were designed using conservative specificity parameters in Primer3 v4.1.0 [40], and in silico validation was performed to minimize confident off-target amplification within the *T. tambroides* reference assembly. Experimental validation was conducted using genomic DNA from 14 *T. tambra* and 18 *T. tambroides* individuals. Primer sequences and primer-specific annealing temperatures are provided in Table S1. Polymerase chain reaction (PCR) products were analyzed by 1.5% agarose gel electrophoresis. The overall analytical framework is summarized in Fig. 1.

### Phylogenomic analysis based on BUSCO single-copy orthologs

To infer the phylogenetic position of *T. tambra* within Cyprinidae, we performed a BUSCO-based phylogenomic analysis using 11 representative cyprinid genomes (including *T. tambroides*, *Danio rerio*, and other representative cyprinids) as shown in Table S2. BUSCO v5.2.2 [28] was run in genome mode with the actinopterygii_odb10 lineage dataset on each assembly to identify complete single-copy orthologs. The resulting BUSCO output directories were processed with the BUSCO_phylogenomics pipeline (https://github.com/jamiemcg/BUSCO_phylogenomics) using the concatenated supermatrix workflow to extract complete single-copy BUSCO protein sequences, align them with MUSCLE, trim alignments with trimAl (automated1), and concatenate the retained loci into a partitioned supermatrix. The default pipeline settings were used.

BUSCO families that were complete and single copy in at least 80% of the sampled species were retained for the concatenated alignment. The resulting partitioned supermatrix was analyzed in IQ-TREE2 v2.3.6 [41] using partition-aware model selection with ModelFinder and partition merging (-m MFP+MERGE and -rcluster 10). Branch support was assessed using 1,000 ultrafast bootstrap replicates and 1,000 Shimodaira–Hasegawa approximate-likelihood-ratio-test replicates. The resulting tree was rooted on *Onychostoma simum* for visualization and displayed using iTOL v6.9.1 [42].

### Genome-wide similarity and ordination analysis using Mash

Genome-wide similarity among *T. tambra*, *T. tambroides*, and representative cypriniform genomes was estimated using Mash v2.3 [43], a MinHash-based method for rapid whole-genome comparison based on shared *k*-mer content. For each assembled genome, Mash sketches were generated using a *k*-mer size of 21 and a sketch size of 500,000 hashes. The *k*-mer length was selected to provide sufficient resolution for large eukaryotic genomes, while the increased sketch size was used to enhance stability and reduce variance in distance estimates among closely related taxa. All other parameters were kept at default settings.

Pairwise Mash distances were computed between all genome sketches to generate a symmetric distance matrix. Mash distance (*D*) approximates the proportion of nucleotide differences between genomes under a Poisson mutation model and provides an alignment-free estimate of genome-wide divergence. Whole-genome similarity was calculated as 1 − *D* to facilitate intuitive interpretation of similarity patterns. The number of shared hashes between genome pairs was also recorded as an indicator of *k*-mer overlap strength.

The resulting pairwise Mash distances were converted into a square matrix and transformed into a whole-genome similarity matrix. This matrix was visualized as a heatmap to illustrate hierarchical genomic relationships among taxa. For clarity of interpretation, *T. tambra* (male and female assemblies) and *T. tambroides* were positioned adjacently in the matrix to allow direct comparison of interspecific similarity relative to other cypriniform species.

To visualize genome-wide divergence in reduced dimensional space, principal coordinates analysis was performed on the Mash distance matrix. The distance matrix was double-centered and subjected to eigen decomposition to obtain principal coordinate axes. The first 2 principal components were retained for visualization, and the proportion of variance explained by each axis was calculated from the corresponding eigenvalues. Ordination patterns were interpreted in the context of established cypriniform phylogenetic relationships.

Mash-based genome comparison captures global *k*-mer similarity across entire assemblies and is independent of gene annotation or alignment-based methods [43]. Although Mash distances are not directly equivalent to alignment-based average nucleotide identity values for large eukaryotic genomes, they provide a rapid and robust estimate of genome-wide relatedness suitable for comparative genomic and phylogenetic context.

### Mitochondrial phylogenetic analysis based on complete mitogenomes

A curated dataset of 15 complete mitochondrial genome sequences representing *T. tambra* and *T. tambroides* was compiled from GenBank for phylogenetic analysis. The final dataset comprised 9 *T. tambra* accessions (KJ880044.1, OK442459.1, OK505795.1, MW471073.1, MW471074.1, MW471075.1, MW471076.1, MW471077.1, and MW471078.1) and 6 *T. tambroides* accessions (MW471068.1, MW471069.1, MW471070.1, MW471071.1, MW471072.1, and PZ137761.1). Only complete mitochondrial genome records were retained, whereas transcriptome records, partial mitochondrial sequences, duplicate accessions, and incomplete or lower-confidence entries were excluded. Available metadata, including isolate name, voucher information, sampling locality, country, tissue source, and collection details, were extracted from GenBank records to document the dataset. Complete mitochondrial genomes were aligned using MAFFT with the --auto and --adjustdirectionaccurately options. A maximum-likelihood phylogeny was then inferred in IQ-TREE2 using ModelFinder for model selection, 1,000 ultrafast bootstrap replicates, and branch-length optimization under the --bnni option.

### Data availability

The draft genome assemblies generated in this study are available in the NCBI under BioProject PRJNA955134 (female; WGS accession JARWAE000000000) and BioProject PRJNA955018 (male; WGS accession JARWAD000000000). Public *T. tambroides* RNA-seq data used for annotation were retrieved from Sequence Read Archive accession SRR14520879. Assembly accessions for comparative genomes used in phylogenomic and Mash analyses are listed in Table S2. Previously published *T. tambra* Illumina short-read data reused in this study were obtained from our previous study [24].

## Results and Discussion

### Genome characterization of male and female *T. tambra*

The genomic sequencing and assembly metrics for male and female *T. tambra* individuals are summarized in Table 1 For the male specimen, the total assembled genome length was 1,250,616,913 bp (1.251 Gb) across 4,139 contigs, with a longest contig of 5,838,465 bp and a GC content of 37.29%. The female assembly had a total length of 1,244,113,601 bp (1.244 Gb) across 3,797 contigs, with a longest contig of 7,635,622 bp and a GC content of 37.28%. These results indicate broadly comparable genome sizes and base composition between the 2 assemblies, with only minor differences in contiguity. The male assembly was approximately 6.5 Mb larger than the female assembly, corresponding to only ~0.5% of the total genome size. This small difference may reflect minor assembly variation, repeat representation, or individual-specific structural differences rather than clear evidence of sex-chromosome differentiation [44,45]. At present, this difference alone does not provide direct evidence for differentiated sex chromosomes or a specific sex-determination system in *T. tambra* [46–48]. Confirmation of structural or chromosomal sex differentiation would require cytogenetic analyses, multi-individual genomic comparisons, and population-level validation [24,49–51].

**Table 1.** Overview of genomic sequencing and assembly results

Parameters	Male	Female
Total contig length (bp)	1,250,616,913	1,244,113,601
Number of contigs	4,139	3,797
Longest contig length (bp)	5,838,465	7,635,622
N50	741,319	758,974
L50	443	425
GC content (%)	37.29	37.28
Completeness (%)	96.2	96.2

### Gene prediction and functional annotation

A total of 50,705 and 49,865 putative protein-coding genes were predicted in the male and female *T. tambra* genomes, respectively. The completeness of the assemblies and predicted gene sets was supported by BUSCO scores of 96.2% for both genomes, with the full breakdown of complete single-copy, duplicated, fragmented, and missing BUSCO categories provided in Table S3. This supports the robustness and reliability of the BRAKER2-based annotation pipeline. Functional annotation using eggNOG-mapper v2 against the eggNOG v5.0 database yielded extensive coverage across multiple functional hierarchies, including COG, KEGG pathways, and Protein Families domains, supporting the accuracy and completeness of the predicted proteomes (Table S4). The overall annotation coverage and functional composition are comparable to those reported for other teleost genomes, including *Cyprinus carpio* [52], *D. rerio* [53], and *T. tambroides* [19].

Although the total number of predicted protein-coding genes in *T. tambra* (~50,000) is higher than that reported for many diploid teleost genomes, this should not be interpreted as evidence of recent whole-genome duplication or polyploidy [47]. Instead, elevated gene counts in large cyprinid genomes are frequently associated with expansive genome size, high repetitive content, complex gene structures, and increased annotation sensitivity when RNA-seq evidence is incorporated [47]. Comparable gene numbers have been reported for *C. carpio* and *T. tambroides*, suggesting that annotation strategy and genome architecture, rather than ploidy alone, contribute substantially to predicted gene counts in mahseer genomes [47]. Future chromosome-level assemblies and synteny-based analyses will further clarify gene copy number variation and genomic organization within the genus *Tor*.

**Fig. 2.**
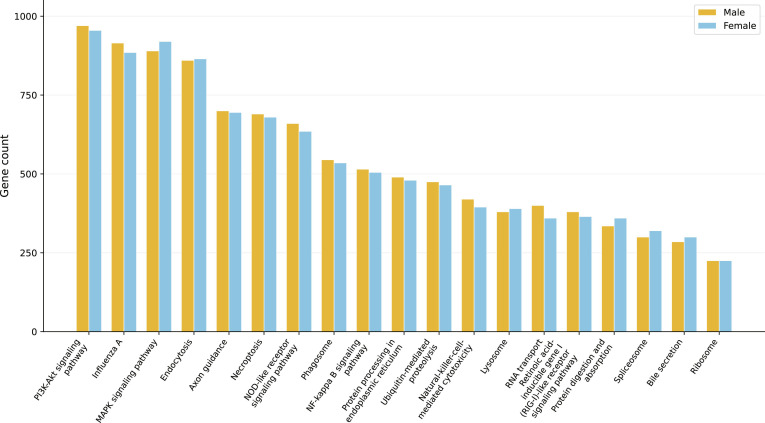
Top 20 Kyoto Encyclopedia of Genes and Genomes (KEGG) pathway categories in male and female *T. tambra* genomes. Comparative distribution of annotated genes across the 20 most enriched KEGG pathways identified in the male and female *T. tambra* genomes. Both sexes exhibited nearly identical functional profiles, dominated by pathways related to signal transduction (phosphatidylinositol 3-kinase-protein kinase B [PI3K-Akt], mitogen-activated protein kinase [MAPK], and nuclear factor kappa B [NF-κB] signaling), immune response (nucleotide-binding oligomerization domain [NOD]-like receptor signaling, phagosome, and necroptosis), protein processing (endocytosis and endoplasmic-reticulum-associated pathways), and RNA transport (retinoic acid-inducible gene I [RIG-I]-like receptor signaling pathway. The consistent enrichment across sexes highlights the conserved regulatory and metabolic complexity of *T. tambra*, reflecting a stable genomic basis for cellular signaling, defense, and developmental processes.

The distribution of genes across COG functional categories was highly similar between male and female genomes (Fig. 2 and Table S5), indicating strong functional conservation between sexes. The largest proportion of annotated genes belonged to category S (Function unknown), accounting for approximately 39% of annotated sequences in both assemblies, followed by category T (Signal transduction mechanisms) and category K (Transcription). This distribution reflects the prominence of regulatory, signaling, and transcriptional control functions within the *T. tambra* genome.

Metabolism-related COG categories, including E (Amino acid transport and metabolism), G (Carbohydrate transport and metabolism), and C (Energy production and conversion), were well represented in both genomes, highlighting the broad metabolic capacity and physiological adaptability of *T. tambra* to diverse freshwater environments. The overall COG distribution closely mirrors patterns reported for other cyprinid fishes, suggesting strong conservation of core functional gene repertoires across the family. The substantial proportion of genes assigned to category S (Function unknown), a feature commonly observed in nonmodel teleost genomes, likely reflects the presence of lineage-specific or rapidly evolving genes that remain poorly characterized [54]. This observation underscores the need for continued expansion of fish genomic resources to improve functional annotation resolution in comparative studies.

KEGG pathway annotation identified 18,658 genes in the male genome and 18,533 genes in the female genome associated with metabolic and regulatory pathways (Fig. 2). The top 20 enriched KEGG pathways showed nearly identical distributions between sexes, indicating highly conserved functional repertoires at the pathway level.

Pathways related to signal transduction and immune response were among the most highly represented, indicating the importance of cellular signaling and defense mechanisms in the biology of *T. tambra*. In addition, pathways associated with endocytosis and protein processing in the endoplasmic reticulum highlight conserved processes involved in protein turnover, cellular homeostasis, and tissue differentiation. Similar functional profiles have been reported in other teleost species, including *Osteoglossum bicirrhosum* [55] and *C. carpio* [52], where core metabolic and signaling pathways dominate the functional landscape.

Overall, the close correspondence between male and female functional annotations suggests that sex-based genomic differentiation in *T. tambra* is unlikely to involve large-scale divergence in gene content or functional pathways, and instead may be driven primarily by localized structural variation or regulatory differences. Taken together, the COG and KEGG analyses reveal a highly conserved and functionally versatile genomic architecture, consistent with the adaptive flexibility and ecological resilience of *T. tambra* in diverse freshwater habitats.

### Microsatellite identification and transposable element/repeat composition

**Fig. 3.**
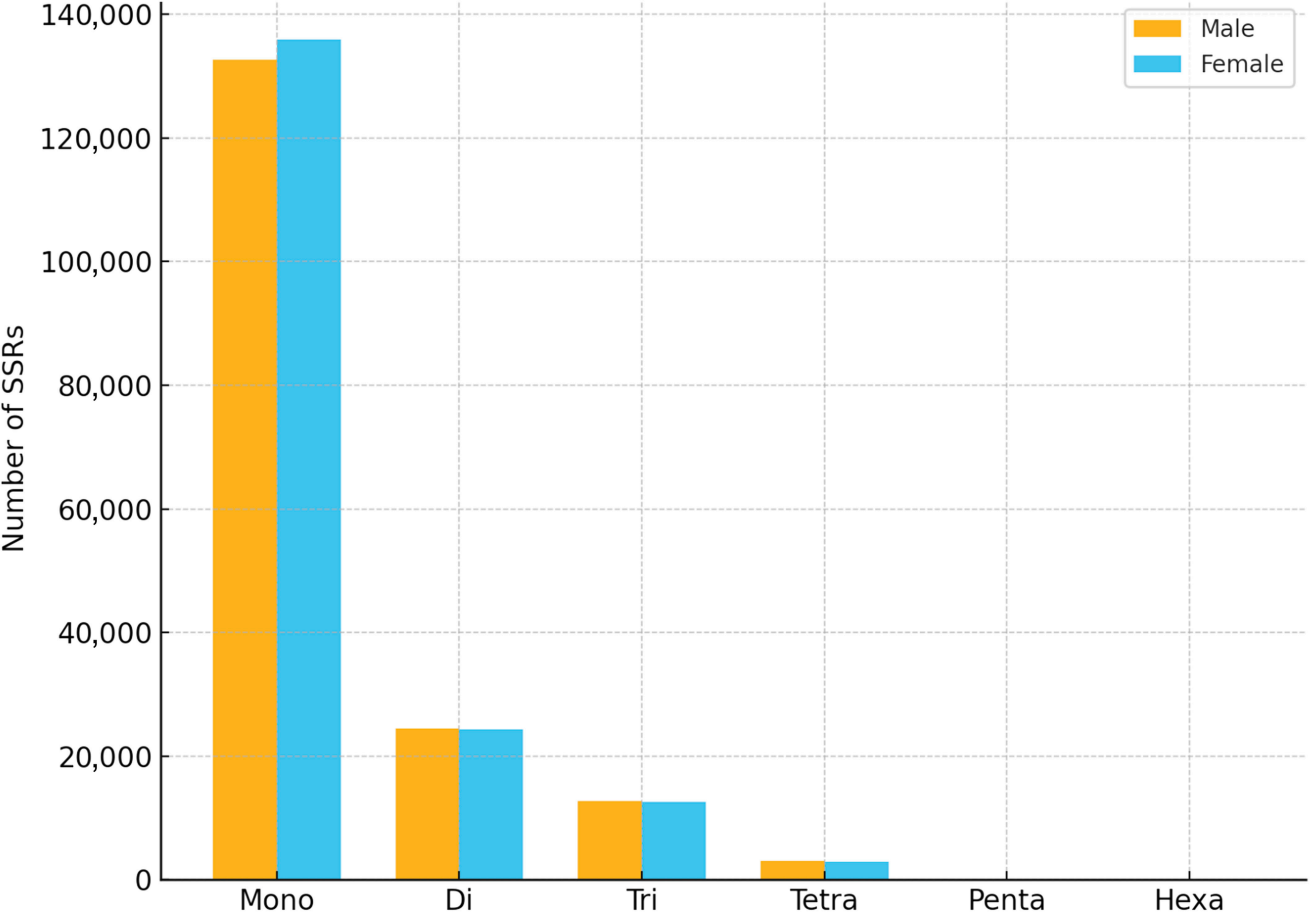
Distribution of simple sequence repeat (SSR) types (mono–hexa) identified in male and female *T. tambra* genomes.

Microsatellites, or simple sequence repeats (SSRs), are ubiquitous, highly polymorphic DNA elements widely distributed throughout eukaryotic genomes, including teleost fishes. They are valuable molecular markers for genetic diversity studies, population structure assessment, parentage analysis, and the construction of genetic linkage maps [56,57]. Microsatellite analysis of the *T. tambra* genomes revealed a high abundance of SSRs in both male and female individuals. In the female genome, a total of 175,958 SSRs were identified across 3,797 contigs spanning approximately 1.244 Gb. Similarly, the male genome contained 172,934 SSRs across 4,139 contigs with a total size of 1.250 Gb. The number of sequences containing SSRs was slightly higher in the male assembly (4,013 sequences) compared to the female assembly (3,754 sequences). Additionally, a substantial number of sequences in both sexes contained multiple SSRs, 3,859 in males and 3,663 in females as shown in Fig. 3 and Table S6.

Regarding repeat type composition, mononucleotide repeats (1-bp unit size) were the most abundant class in both genomes, accounting for 136,017 SSRs (77.3%) in females and 132,685 SSRs (76.7%) in males. Dinucleotide (2 bp) repeats were the second most prevalent, followed by trinucleotide (3 bp) repeats. The frequency of tetranucleotide, pentanucleotide, and hexanucleotide repeats decreased progressively in both sexes. Compound SSRs, where multiple microsatellite motifs occur adjacently or interrupted by short sequences, were also identified in both genomes, comprising 7,494 in females and 7,377 in males. The distribution patterns of SSR types were highly similar between the male and female *T. tambra* assemblies, indicating consistent SSR class composition within the species.

This finding aligns closely with patterns observed in other cyprinid species, where mononucleotide repeats consistently represent the predominant SSR type. For instance, a recent study on the genome of *T. tambroides* [19], a closely related mahseer species, reported that mononucleotide SSRs accounted for approximately 75% to 80% of all microsatellites, with A/T motifs being particularly prevalent [19]. Such dominance of mononucleotide repeats is a common feature in teleost genomes and is often attributed to their high mutability and abundance in noncoding genomic regions [58,59].

The relative proportions of dinucleotide and trinucleotide repeats in *T. tambra* also exhibited a pattern typical of cyprinid fishes. Dinucleotide repeats comprised around 13.8% to 14.2% of the total SSRs, while trinucleotide repeats accounted for approximately 7.1% to 7.3%. Similar distributions have been reported in the common carp (*C. carpio*), where mononucleotide repeats constitute about 70% to 75% of total SSRs, with dinucleotide and trinucleotide repeats representing substantial proportions of the remainder [52]. Interestingly, in zebrafish (*D. rerio*), while mononucleotide repeats remain predominant, there is a slightly higher relative abundance of trinucleotide and tetranucleotide repeats. This is likely a reflection of the highly curated and gene-dense nature of the zebrafish genome, where trinucleotide repeats frequently occur within coding regions without disrupting the reading frame [53].

The predominance of mononucleotide repeats and the substantial presence of di- and trinucleotide SSRs indicate that the overall SSR class composition of *T. tambra* is broadly similar to patterns reported in other cyprinid fishes, including *T. tambroides*, *C. carpio*, and *D. rerio*. However, the present analysis did not assess whether corresponding SSRs were located in homologous sequences or conserved at the same genomic loci between assemblies or across species. A more detailed motif-level and locus-level comparison would therefore be valuable in future studies. These findings nevertheless provide a useful foundation for developing species-specific microsatellite markers for genetic diversity assessment, population structure analysis, and conservation management.

De novo repeat annotation revealed substantial repetitive content in both male and female *T. tambra* genomes and showed that the 2 assemblies have broadly similar repeat landscapes (Table S7). In total, 46.94% of the female genome and 46.91% of the male genome were masked as repetitive DNA. Among these, retroelements accounted for 6.78% and 6.76%, respectively, whereas DNA transposons accounted for 4.78% and 4.75%. A substantial proportion of the repetitive fraction remained unclassified (32.53% in female; 32.55% in male), while simple repeats accounted for 2.19% and 2.20% and low-complexity regions for 0.29% in both assemblies. The classified custom repeat library recovered recognizable transposable element (TE)-related families from multiple subclasses, including DNA transposons, long-interspersed-nuclear-element-related elements, and long-terminal-repeat-related elements, with examples such as DNA/TcMar-Tc1, DNA/Kolobok-T2, DNA/hAT-Ac, and long interspersed nuclear element/Rex-Babar, although many repeat families remained classified as unknown. This pattern indicates that the *T. tambra* genome contains substantial lineage-specific or incompletely characterized repetitive content. Repeat-associated sequence turnover may therefore contribute to some of the localized divergent regions detected by the coverage-based marker-discovery framework and may partly explain the reduced specificity observed for many candidate primer pairs. For contextual comparison, a previous genome study of *T. tambroides* also reported substantial repetitive content and a diverse TE complement, including 39.84% total interspersed repeats, with DNA transposons as the largest category (20.33%) followed by unclassified repeats (10.11%). However, direct quantitative comparison should be interpreted cautiously because the assemblies and repeat-annotation workflows differed.

### *K*-mer-based identification of sex-enriched genomic sequences

Given that only a single individual per sex was analyzed, this analysis is exploratory and intended to generate hypotheses rather than provide definitive evidence of sex-linked genomic architecture. To explore the presence of sex-associated genomic signals in *T. tambra*, an assembly-free *k*-mer-based comparative analysis was conducted using whole-genome sequencing reads from one male and one female individual. Genome-wide approaches for identifying sex-associated loci have proven effective in nonmodel fish species, where sex-determination systems are often complex and poorly resolved [60,61]. *K*-mers were counted using KMC under increasingly stringent parameters to minimize the effects of sequencing errors and stochastic variation.

At lower stringency thresholds (*k* = 21; minimum count ≥3 and ≥10), tens of millions of apparent sex-specific *k*-mers were detected in both individuals, indicating that relaxed filtering is strongly influenced by individual polymorphism and coverage heterogeneity. Increasing stringency to *k* = 31 with a minimum count threshold ≥20 substantially reduced low-confidence *k*-mers and revealed that approximately 94% of high-confidence *k*-mers were shared between male and female datasets, consistent with a largely conserved genomic background.

When focusing on moderate-copy *k*-mers (200 to 499 occurrences), a pronounced asymmetry emerged (Fig. S1A and B). The female dataset contained 380,540 female-specific *k*-mers compared with 41,219 male-specific *k*-mers, representing an approximately 9-fold enrichment of female-associated sequences. Similar enrichment patterns have been reported in genome-wide studies identifying sex-linked loci and sex-associated genomic regions in teleost fishes [62]. Additional validation using a subset of the 5,000 highest-abundance female-specific *k*-mers confirmed their absence from the male *k*-mer database under stringent filtering criteria, supporting a female-biased sequence signal.

However, this analysis was conducted using only a single male and a single female individual. Therefore, the observed enrichment pattern should be interpreted cautiously. While a female-biased *k*-mer signal was detected under stringent filtering criteria, such asymmetry may arise from sex-associated genomic regions, but it may also reflect nonbiological or individual-level factors such as coverage heterogeneity between datasets, differences in library complexity, individual repeat-content variation, individual-specific structural differences, or sampling effects. Notably, sex-determination systems in fish frequently exhibit rapid evolutionary turnover and complex genetic architectures, often involving multiple loci and environmental interactions [60,63]. Validation across multiple individuals and independent populations will be necessary to determine whether these *k*-mer differences represent stable sex-linked genomic features in *T. tambra*. Because only single representatives of each sex were analyzed, these findings should be considered preliminary and hypothesis generating rather than definitive evidence of sex chromosome differentiation.

### Phylogenomic analysis based on BUSCO single-copy orthologs

A genome-scale phylogenomic analysis was conducted using complete single-copy orthologs identified with BUSCO under the actinopterygii_odb10 lineage dataset. BUSCO analyses were performed independently for each of the 11 representative cypriniform taxa, including *T. tambra* and *T. tambroides*, under identical parameters to ensure comparability across assemblies.

**Fig. 4.**
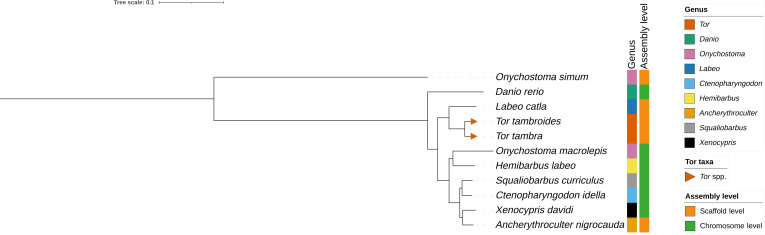
Maximum-likelihood phylogeny of 11 cypriniform taxa inferred from a partitioned BUSCO supermatrix. The displayed tree was rooted on *O. simum*. Branch lengths represent substitutions per site. Node labels show Shimodaira–Hasegawa approximate likelihood ratio test/ultrafast bootstrap support values. The sampled *T. tambra* and *T. tambroides* genomes form a closely related lineage within *Tor*.

Across all taxa, 3,625 unique BUSCO ortholog IDs were identified as complete and single copy in at least one species. After applying a ≥90% occupancy threshold, 1,653 loci were retained, yielding a partitioned supermatrix of 926,415 aligned amino acid positions across 11 taxa. Maximum-likelihood analysis of this dataset recovered *T. tambra* and *T. tambroides* as 2 distinct but closely related lineages forming a monophyletic *Tor* clade (Fig. 4). The node separating the 2 taxa received strong support, indicating a consistent genome-scale nuclear phylogenetic signal differentiating these sampled lineages.

Branch lengths between *T. tambra* and *T. tambroides* were short relative to deeper divergences among other cypriniform taxa, consistent with recent divergence and/or incomplete lineage sorting. Such patterns are characteristic of recently diverged lineages, where ancestral polymorphisms persist across speciation events and reduce apparent genetic distances despite genome-wide differentiation [64]. The recovery of well-supported monophyletic lineages despite these short interspecific branch lengths indicates that phylogenetic signal is distributed across a large number of independent nuclear loci, allowing the concatenated supermatrix to overcome stochastic variation at individual genes.

Importantly, the strong bootstrap support observed across the phylogeny suggests that the inferred relationships are not driven by a limited subset of highly informative loci but instead reflect concordant signal across hundreds to thousands of conserved nuclear genes. This pattern is consistent with expectations from genome-scale phylogenomics, where increasing locus number improves both resolution and statistical confidence, particularly for shallow evolutionary splits [64].

Despite their morphological similarity and the limited resolution often observed with mitochondrial markers, the nuclear genome-scale dataset robustly resolved the 2 taxa as independent evolutionary lineages. This contrast highlights the inherent limitations of mitochondrial DNA, which represents a single maternally inherited locus and is therefore more susceptible to stochastic lineage sorting, introgression, and selective sweeps [65]. In contrast, nuclear phylogenomic datasets integrate signal across numerous independently segregating loci, providing a more comprehensive and reliable representation of species history [64].

Beyond the *Tor* clade, the phylogeny recovered relationships consistent with established cypriniform systematics [66], including the placement of *Ctenopharyngodon idella* within derived cyprinids and the separation of *D. rerio* from more crownward lineages. Most internal nodes exhibited strong bootstrap support, demonstrating that the BUSCO-based supermatrix provides sufficient genome-wide signal to resolve both shallow and deeper evolutionary splits with high confidence.

However, because only one representative genome per focal species was included, and only one public *T. tambroides* genome assembly was available at the time of analysis, this phylogenomic reconstruction does not by itself provide a population-level test of species monophyly across the geographic ranges of *T. tambra* and *T. tambroides*. Broader sampling from multiple populations and additional genome assemblies will be necessary to further evaluate species boundaries and confirm whether the observed lineage-level separation is consistently maintained across natural populations.

Collectively, these results indicate that the sampled *T. tambra* and *T. tambroides* genomes are separable as distinct nuclear lineages despite their high overall sequence similarity. Because the phylogenomic analysis integrates 1,653 conserved single-copy nuclear loci, the observed separation does not depend on a single marker or a few exceptional genes. Rather, multiple independent nuclear loci provide concordant signal supporting differentiation between these sampled lineages. However, because only one representative genome per focal species was included, this analysis does not by itself provide a population-level test of species monophyly across the geographic ranges of *T. tambra* and *T. tambroides*. Broader sampling from multiple populations will be necessary to further evaluate species boundaries and confirm whether the observed lineage-level separation is consistently maintained across natural populations. This result also does not imply uniformly elevated divergence across the genome; instead, it indicates that genome-scale nuclear data recover consistent lineage-level signal even though the strongly differentiated regions identified by the coverage-based marker-discovery framework are limited and localized.

### Genome-wide similarity analysis of *Tor* species within Cypriniformes

Mash-based genome comparison showed highly consistent whole-genome similarity patterns among the sampled cypriniform genomes. The male and female *T. tambra* assemblies showed near-identical distances to *T. tambroides* (male: *D* = 0.01514; female: *D* = 0.01515; *P* ≈ 0), corresponding to approximately 98.48% to 98.49% genome-wide *k*-mer similarity (≈285,800 shared hashes out of 500,000). These highly similar estimates indicate that the inferred interspecific divergence is stable and not attributable to sex-specific assembly artifacts or stochastic variation in genome reconstruction.

**Fig. 5.**
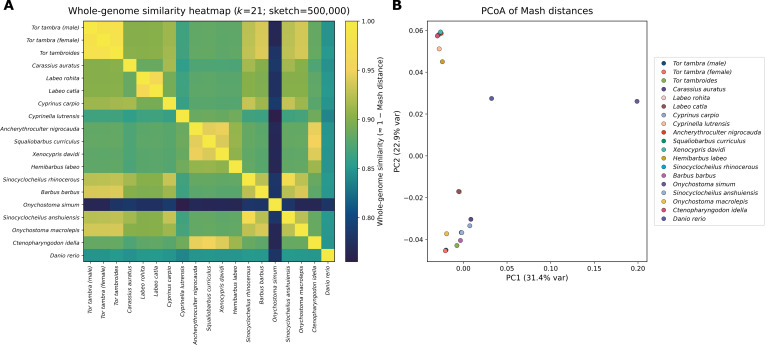
Whole-genome similarity and ordination analysis based on Mash distances among cypriniform genomes. (A) Heatmap of whole-genome similarity estimated as 1 − Mash distance (*k* = 21; sketch size = 500,000). Higher values indicate greater genome-wide *k*-mer similarity. *T. tambra* (male and female assemblies) cluster closely with *T. tambroides*, showing the highest pairwise similarity within the dataset, while other cypriniform taxa exhibit progressively lower similarity. (B) Principal coordinates analysis (PCoA) derived from the Mash distance matrix. PC1 and PC2 explain 30.7% and 23.3% of the total variance, respectively. The 2 *T. tambra* assemblies cluster tightly and group nearest to *T. tambroides*, consistent with recent divergence and high genome-wide similarity relative to other cypriniform species.

To place this divergence in a broader evolutionary context, we expanded the Mash analysis to include representative cypriniform genomes. The whole-genome similarity heatmap (Fig. 5A) revealed a clear hierarchical structure, with the 2 *T. tambra* assemblies clustering tightly together and showing the highest similarity to *T. tambroides*. In contrast, other cypriniform taxa—including *Cyprinella lutrensis*, *Sinocyclocheilus anshuiensis*, *Sinocyclocheilus rhinocerous*, *Cyprinus carpio*, *Labeo rohita*, *Labeo catla*, *Ctenopharyngodon idella*, and *Danio rerio*—exhibited progressively lower similarity values relative to the *Tor* clade. This pattern is consistent with established phylogenetic relationships within Cypriniformes [66].

Principal coordinates analysis (Fig. 5B) further resolved these relationships in reduced dimensional space. The 2 *T. tambra* assemblies formed a tight cluster and grouped immediately adjacent to *T. tambroides* along the first 2 principal axes (PC1 = 30.7% of variance; PC2 = 23.3%). Species from other cypriniform lineages occupied increasingly distant positions in ordination space, with *Sinocyclocheilus* spp., *Cyprinella lutrensis*, and *C. carpio* showing intermediate divergence, and more distantly related taxa such as *D. rerio* separating further along PC1. The ordination therefore recapitulates known evolutionary relationships and demonstrates that genome-wide *k*-mer similarity captures phylogenetic structure across the dataset.

Although overall genomic similarity between *T. tambra* and *T. tambroides* is high, the measurable whole-genome *k*-mer distance (*D* ≈ 0.015) indicates reproducible assembly-level differences between the taxa. Because Mash summarizes shared *k*-mer content across entire genome assemblies, it provides an aggregate estimate of overall genomic relatedness, but it does not localize where divergent regions occur or quantify the proportion of the genome represented by strongly differentiated loci. Accordingly, the Mash result complements, rather than contradicts, the mapping-based analysis in the “Identification of candidate nuclear markers distinguishing *T. tambra* and *T. tambroides*” section. Under that stringent coverage-based framework, only a small fraction of the reference genome showed persistent coverage asymmetry and zero-coverage intervals suitable for marker development. Thus, the 2 approaches resolve different scales of variation: Mash captures global whole-assembly dissimilarity, whereas the mapping-based framework identifies localized high-contrast candidate regions.

Rather than indicating deep genomic separation, the Mash results highlight a pattern characteristic of closely related but independently evolving taxa: extensive overall sequence similarity coupled with reproducible whole-assembly dissimilarity. The concordant placement of *Tor* species relative to other cypriniform genera further reinforces the phylogenetic coherence of the genus *Tor* within the broader cyprinid radiation. Collectively, these genome-wide analyses demonstrate that while *T. tambra* and *T. tambroides* share a high degree of nuclear genomic similarity consistent with recent divergence, they are nevertheless separable as distinct evolutionary entities based on genome-scale data.

### Identification of candidate nuclear markers distinguishing *T. tambra* and *T. tambroides*

Cross-species alignment of *T. tambra* Illumina reads against the *T. tambroides* reference assembly revealed extensive genome-wide sequence conservation. Across 44,726 scaffolds, the vast majority exhibited high coverage breadth, typically exceeding 95%, with many scaffolds showing 98% to 99% base coverage. Weighted mean sequencing depth reached approximately 40× per individual and 80.6× when male and female datasets were pooled. Overall read mapping rates exceeded 96%, confirming strong cross-species genomic similarity and extensive synteny between the 2 taxa, consistent with the high mapping efficiency expected between closely related genomes.

To evaluate the robustness of the mapping-based estimate of localized divergence, we repeated the zero-coverage analysis using 3 mapping-quality thresholds (mapping quality ≥10, ≥20, and ≥30) for both male and female *T. tambra* read sets aligned to the *T. tambroides* reference genome. Across these settings, weighted mean depth declined modestly with increasing stringency, as expected, but the overall pattern remained stable between sexes. Total zero-coverage sequence represented approximately 2.06% to 2.40% of the reference genome in the male dataset and 2.06% to 2.42% in the female dataset (Table S8). When analysis was restricted to longer zero-coverage intervals (≥500 bp), the corresponding proportion was reduced to approximately 1.02% to 1.35% in males and 1.01% to 1.34% in females. These results indicate that the exact proportion of candidate high-asymmetry regions is parameter dependent, but the conclusion that such regions comprise only a small fraction of the *T. tambroides* reference genome under the present mapping-based framework is robust across reasonable alignment-quality thresholds.

**Fig. 6.**
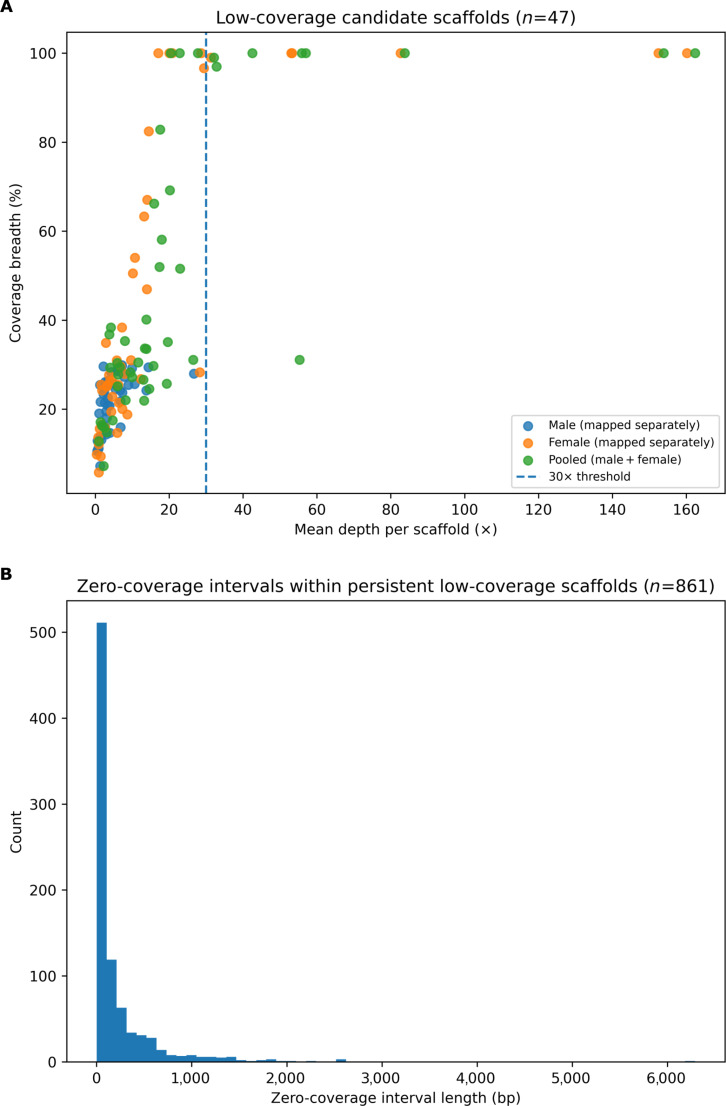
Coverage-based identification of candidate divergent regions between *T. tambra* and *T. tambroides*. (A) Coverage asymmetry among persistent low-depth scaffolds following pooled cross-species read mapping. Mean sequencing depth (×) is plotted against coverage breadth (%) for scaffolds exhibiting persistent low coverage after pooling male and female *T. tambra* reads mapped to the *T. tambroides* reference genome. (B) Length distribution of zero-coverage intervals within persistent low-depth scaffolds. Histogram showing the distribution of zero-coverage intervals identified within the low-depth scaffolds retained after pooled mapping.

When male and female datasets were analyzed separately, a moderate fraction of scaffolds showed reduced mean sequencing depth. Specifically, 5,176 scaffolds (11.6%) in the male dataset and 5,961 scaffolds (13.3%) in the female dataset exhibited mean depth below 30×. However, after pooling reads from both sexes to mitigate stochastic depth variation, the number of scaffolds with mean depth <30× decreased markedly to 293 scaffolds (0.65% of the total assembly). This substantial reduction indicates that most low-depth signals observed in individual datasets primarily reflect sampling variability rather than true absence or divergence (Fig. 6A), consistent with known effects of sequencing depth heterogeneity and coverage bias in short-read datasets [67].

To identify robust candidate-species-associated regions, we focused on scaffolds exhibiting persistently reduced coverage under pooled mapping conditions. Among these, 47 scaffolds displayed mean depth <30× (approximately <0.4× of the normalized genome-wide mean depth) and showed substantially reduced coverage breadth relative to the >98% background observed across conserved scaffolds. Across these 47 scaffolds, mean depth was 23.89× and mean coverage breadth was 48.32%, indicating pronounced and localized divergence.

To further refine candidate loci, zero-coverage intervals were extracted from pooled alignments within the 47 low-depth scaffolds. A total of 861 discrete zero-coverage intervals were identified, spanning 186,476 bp in aggregate, with a mean interval length of 216.6 bp (Fig. 6B). These uncovered segments represent genomic regions within the *T. tambroides* reference lacking read support from *T. tambra*, consistent with localized sequence absence, high divergence, or structural differentiation rather than uniform genome-wide divergence.

Importantly, the persistence of these low-depth scaffolds after pooling, combined with their internal zero-coverage intervals, demonstrates that strongly differentiated regions detectable by this coverage-based framework are highly localized and represent only a small fraction of the reference genome under the applied detection criteria. This pattern is consistent with expectations for recently diverged taxa, in which genomic differentiation is heterogeneous and concentrated in a limited number of regions while the majority of the genome remains conserved [64,68].

Candidate scaffolds were further screened to identify internal regions suitable for primer development, prioritizing segments showing consistent cross-species coverage asymmetry. Experimental validation across multiple individuals per species confirmed that several primer pairs produced reproducible species-specific amplification patterns. In cases where amplification occurred in both species, Sanger sequencing revealed fixed interspecific nucleotide substitutions within the amplified region, further supporting their diagnostic reliability.

Collectively, these results demonstrate that although genome-wide nuclear similarity between *T. tambra* and *T. tambroides* is high, a small fraction of scaffolds (0.65% under pooled mapping) exhibit persistent coverage reduction and localized zero-coverage segments. These localized divergent regions may represent genomic islands of differentiation driven by selection, structural variation, or recombination suppression [69,70], potentially reflecting early-stage genomic divergence following recent speciation.

### Molecular marker development and validation

To further evaluate mitochondrial resolution in the *T. tambra*–*T. tambroides* species pair, we analyzed a curated dataset of 15 complete mitochondrial genomes representing both taxa. The mtDNA phylogeny did not recover clean reciprocal monophyly of *T. tambra* and *T. tambroides*. Although some local lineages formed compact clusters, mitochondrial haplotypes were interspersed between the 2 nominal species, indicating that complete mitochondrial genomes alone provide limited species-level resolution for this comparison. This pattern is consistent with recent divergence and may reflect incomplete lineage sorting, historical introgression, or retention of geographically structured ancestral haplotypes. The complete-mitogenome phylogeny is shown in Fig. S2.

As an additional locus-specific assessment of mitochondrial resolution, a 625-bp fragment of the COX1 gene was analyzed using BLASTN against the NCBI database. The sequence showed 99.76% to 100% identity to *T. tambra* and 100% identity to *T. tambroides*, indicating that COX1 alone lacks sufficient discriminatory power for reliable species-level differentiation between these taxa. This pattern is consistent with previous mitochondrial evidence from our group. In our earlier study, complete mitochondrial genome analysis and phylogenetic reconstruction based on whole mtDNA as well as multiple mitochondrial markers (12S, 16S, ATP6, ATP8, COX1, COX2, COX3, Cytb, ND1, ND2, ND3, ND4, ND4L, ND5, and ND6) also failed to clearly distinguish *T. tambra* from *T. tambroides*, indicating that the limited mitochondrial resolution is not restricted to a single COX1 fragment but reflects a broader mitochondrial pattern in this comparison [24]. More recently, an independent mitochondrial study of Thai *T. tambra* based on COI and Cytb likewise revealed substantial lineage complexity and taxonomic ambiguity within the group, further underscoring that mitochondrial data alone can be difficult to interpret for species-level diagnosis in *Tor* [71]. In contrast, the genome-scale nuclear analyses presented here consistently resolved *T. tambra* and *T. tambroides* as distinct lineages and enabled the identification of candidate diagnostic nuclear loci.

Based on the coverage-based discovery framework, 38 primer pairs (Table S1) were designed targeting candidate nuclear loci exhibiting reduced cross-species coverage or localized zero-coverage intervals. In silico mapping of primer pairs against available reference assemblies suggested species-restricted amplification patterns for several candidates: 6 primer pairs aligned uniquely to the *T. tambroides* reference, whereas 32 primer pairs mapped exclusively to the *T. tambra* reference assembly.

**Fig. 7.**
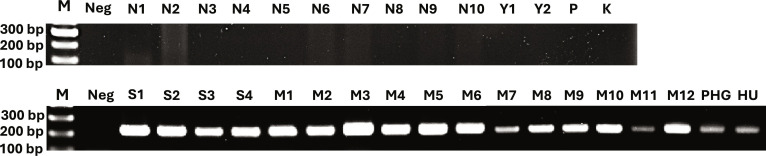
Agarose gel electrophoresis demonstrating species-specific amplification using the primer pair *Tor tambroides*1*_*F and *Tor tambroides*1*_*R. M: 100-bp DNA ladder; Neg: negative control. Lanes N, Y, P, and K correspond to *T. tambra* samples collected from Narathiwat, Yala, Phetchaburi, and Kanchanaburi Provinces (Thailand), respectively. Lanes S, M, PHG, and Hu correspond to *T. tambroides* samples from the Salween River, Myanmar, Endau (Pahang), and Hulu Langat (Selangor), Malaysia.

However, experimental PCR validation revealed that only one primer pair (*T. tambroides*1_F and *T. tambroides*1_R; Table S1) produced a clear species-specific amplification pattern. This primer pair generated a 218-bp amplicon exclusively in *T. tambroides*, with no detectable amplification in *T. tambra* individuals (Fig. 7). The remaining 37 primer pairs produced amplification products in both species. The low proportion of fully species-specific markers reflects the extremely high genomic similarity between the 2 taxa and highlights the stringency of our discovery framework, which prioritizes robustness over overfitting to assembly-specific or mapping artifacts.

The discrepancy between in silico specificity predictions and experimental outcomes likely reflects limitations in reference genome completeness and structural accuracy, particularly for the draft *T. tambroides* assembly available in public databases. In addition, paralogous sequences, repetitive elements, or unannotated homologous regions may have contributed to cross-amplification despite predicted specificity.

In cases where cross-species amplification occurred, Sanger sequencing of PCR products identified fixed interspecific nucleotide substitutions within several loci, indicating that even non-species-specific amplification products may retain discriminatory value at the sequence level.

Collectively, these results demonstrate that while mitochondrial *COX1* sequences are insufficient for resolving *T. tambra* and *T. tambroides*, genome-guided nuclear marker development enables identification of diagnostic loci. Although in silico screening overestimated species specificity, experimental validation confirmed at least one robust species-specific nuclear marker and identified additional loci containing fixed interspecific variation suitable for molecular discrimination.

Consistent with this interpretation, the de novo repeat annotation recovered multiple TE-related families in the *T. tambra* genome, suggesting that repeat-associated sequence turnover may underlie part of the localized coverage asymmetry and low experimental specificity observed among candidate loci.

## Conclusion

This study provides comprehensive genome-scale nuclear evidence supporting species-level differentiation between *T. tambra* and *T. tambroides*, despite their high overall genomic similarity and the limited resolution of mitochondrial markers. Phylogenomic reconstruction based on conserved single-copy BUSCO orthologs robustly resolved both taxa as distinct evolutionary lineages, while Mash-based genome-wide comparisons demonstrated reproducible whole-genome dissimilarity between the assemblies. Under the applied coverage-based marker-discovery framework, strongly differentiated candidate regions were highly localized and represented only a small fraction of the reference genome. Importantly, the validated diagnostic marker is supported by multi-individual PCR testing, whereas the inferred genomic extent of localized divergence remains dependent on the present single-reference comparative framework and limited whole-genome sampling. These regions provided a reliable basis for the development of diagnostic nuclear markers. Experimental validation confirmed at least one robust species-specific marker and identified additional loci containing fixed interspecific variation. Together, these findings demonstrate that genome-wide nuclear approaches substantially improve resolution for species delimitation in recently diverged taxa and provide a reproducible framework for marker discovery. This work has direct implications for taxonomic clarification, conservation genetics, and broodstock management of mahseer species across Southeast Asia.

## Data Availability

The draft genome assemblies generated in this study are available in the NCBI under BioProject PRJNA955134 (female; WGS accession JARWAE000000000) and BioProject PRJNA955018 (male; WGS accession JARWAD000000000). Public *Tor tambroides* RNA-seq data used for annotation were retrieved from Sequence Read Archive accession SRR14520879. Assembly accessions for comparative genomes used in phylogenomic and Mash analyses are listed in Table S2. Previously published *Tor tambra* Illumina short-read data reused in this study were obtained from our previous study [24].

## Supplementary Materials

Supplementary 1 Tables S1 to S8 Figs. S1 and S2 Scripts S1 and S2
