# Supplementary material for: Genome-Scale Nuclear Markers Provide Strong Evidence for Species-Level Differentiation between the Mahseer Fishes Tor tambra and Tor tambroides
Source: Comput Struct Biotechnol J. 2026 May 28;35(1):0112. doi: 10.34133/csbj.0112 (PMC13216894; doi:10.34133/csbj.0112)
Supplement: Supplementary 1 — Tables S1 to S8 Figs. S1 and S2 Scripts S1 and S2 [file csbj.0112.f1.docx]

**Supplementary** **materials**

**Table S1.** Primer sequences used in this study, including their corresponding annealing temperatures and annealing times for PCR amplification.

| **No.** | **Primer name** | **Primer sequence** | **Annealing temp. and time** | **Expect size** |
| --- | --- | --- | --- | --- |
| 1 | *T. tambroides*1_F | ACCTTTCTGCAGCCTTGTGT | 60°C,30 s. | 218 bp. |
|  | *T.* tambroides1_R | AGTTGGCTGAACGATGCAGA |  |  |
| 2 | *T. tambroides*2_F | TGCACTGTTGTTGTCCAAAC | 60°C,30 s. | 238 bp. |
|  | *T. tambroides*2_R | TCGACTGTCACAGAAACGCA |  |  |
| 3 | *T. tambroides*3_F | GTGAGCTACTGCAGACCTCA | 60°C,30 s. | 233 bp. |
|  | *T. tambroides*3_R | TCAGTCATTGAGCAGCATGTCT |  |  |
| 4 | *T. tambroides*4_F | GCTTTGTGTACTGTGGCTGC | 60°C,30 s. | 481 bp. |
|  | *T. tambroides*4_R | CCATATGAGTGGGGAAGGGC |  |  |
| 5 | *T. tambroides*5_F | ACTCGTTTCCAGGCACTCTG | 57°C,35 s. | 307 bp. |
|  | *T. tambroides*5_R | CAGCAAGGACCGGGAAATCT |  |  |
| 6 | *T. tambroides*6_F | GCTCTGGATCAGGGGTTGTC | 60°C,30 s. | 354 bp. |
|  | *T. tambroides*6_R | AGACTTGTCTGCGGCATTCA |  |  |
| 7 | *T. tambra*1_F | GTCCTCGTCTTCAAAGCCGA | 62°C,30 s. | 210 bp. |
|  | *T. tambra*1_R | CCCCTACAGCAGACTTTGGG |  |  |
| 8 | *T. tambra*2_F | AACTGCAGCAATGAAGCAGC | 60°C,30 s. | 254 bp. |
|  | *T. tambra*2*_*R | CTAGAGCACTGCAGCACTGT |  |  |
| 9 | *T. tambra*3_F | CCAGTCCAGCCAGTTTTTGC | 62°C,30 s. | 303 bp. |
|  | *T. tambra*3_R | CGCGGGTGACAAGCTATTCT |  |  |
| 10 | *T. tambra*4_F | AACTATGCTAGCCCTGCCAC | 62°C,30 s. | 443 bp. |
|  | *T. tambra*4_R | TGTCCCATGAACTGCACAGG |  |  |
| 11 | *T. tambra*5_F | ACCAACGTCTAGCAGCAGAC | 62°C,30 s. | 218 bp. |
|  | *T. tambra*5_R | CGGGTGAGTGTGTGATGACA |  |  |
| 12 | *T. tambra*6_F | TGTCCCATGAACTGCACAGG | 62°C,30 s. | 443 bp. |
|  | *T. tambra*6_R | AACTATGCTAGCCCTGCCAC |  |  |
| 13 | *T. tambra*7_F | CCATTGCCCATCAGGTGTCT | 63°C,30 s. | 350 bp. |
|  | *T. tambra*7_R | AGCCCAAAACCCACAGAACA |  |  |
| 14 | *T. tambra*8_F | ACAGTAGTTTGCCATGCCGA | 61°C,30 s. | 484 bp |
|  | *T. tambra*8_R | TACAAGAGCTGAATGGCGCA |  |  |
| 15 | *T. tambra*9_F | CTGTTCGCGCCTCAATTACG | 61°C,30 s. | 349bp. |
|  | *T. tambra*9_R | TGCTAGGTCTTGCCACAGTG |  |  |
| 16 | *T. tambra*10_F | TCCATCCATTTCCACAGGCC | 62°C,30 s. | 308 bp. |
|  | *T. tambra*10_R | AGCACTGCATCCATGTCGAA |  |  |
| 17 | *T. tambra*11_F | ACCACCTGACGAAGAACGAC | 62°C,30 s. | 492 bp. |
|  | *T. tambra*11_R | CTATCGGTGAGAGCACGTCC |  |  |
| 18 | *T. tambra*12_F | ACCGATGGGAGGGAAGATGA | 63°C,30 s. | 879 bp. |
|  | *T. tambra*12_R | TGGGACATCGCCTAGTCTGA |  |  |
| 19 | *T. tambra*13_F | CAAAACTGAACCGCCCACAG | 62°C,30 s. | 915 bp. |
|  | *T. tambra*13_R | TCAGAAGCCACGTCTGTGTC |  |  |
| 20 | *T. tambra*14_F | GTGTGCACCTTCTGTAGCCA | 63°C,35 s. | 700 bp. |
|  | *T. tambra*14_R | TCTGGTGCTCAGAGCTCTGT |  |  |
| 21 | *T. tambra*15_F | CACAGGTACGCTTCACCACT | 62°C,30 s. | 889 bp. |
|  | *T. tambra*15_R | CCTGGTCACACTTCAGCTGT |  |  |
| 22 | *T. tambra*16_F | TGGATTCAAGGGCAGTGACC | 62°C,30 s. | 889 bp. |
|  | *T. tambra*16_R | CGTGACGTACAAGGGACCAA |  |  |
| 23 | *T. tambra*17_F | GGTGTCCACTCTGACCCATG | 63°C,30 s. | 828 bp. |
|  | *T. tambra*17_R | ACAGGGGGAAAACGGTTGTT |  |  |
| 24 | *T. tambra*18_F | TTGGTCCTTGTACGTCCACG | 59°C,35 s. | 860 bp. |
|  | *T. tambra*18_R | ATCTGTGGGAGGGAGGAGAC |  |  |
| 25 | *T. tambra*19_F | GTCGTTCTTCGTCAGGTGGT | 62°C,30 s. | 985 bp. |
|  | *T. tambra*19_R | AGACAGAGGAGCACGCAAAA |  |  |
| 26 | *T. tambra*20_F | GGTGTCCACTCTGACCCATG | 63°C,30 s. | 828 bp. |
|  | *T. tambra*20_R | ACAGGGGGAAAACGGTTGTT |  |  |
| 27 | *T. tambra*21_F | ATTCACTGCATTGCCCTCCA | 60°C,30 s. | 751 bp. |
|  | *T. tambra*21_R | GACGTGCTCTCACCCGATAG |  |  |
| 28 | *T. tambra*22_F | GCCTCATTCAGTCCTCTCGG | 63°C,30 s. | 897bp. |
|  | *T. tambra*22_R | CGTGGACGTACAAGGACCAA |  |  |
| 29 | *T. tambra*23_F | GACAGAGTGGACGGCGATAG | 62°C,30 s. | 773 bp. |
|  | *T. tambra*23_R | GGAGATTCAGCCAGGCAGTT |  |  |
| 30 | *T. tambra*24_F | TTCAAGACGGGGCATTGAGA | 63°C,30 s. | 850 bp. |
|  | *T. tambra*24_R | TGACGCTGAGATGAGGGGTA |  |  |
| 31 | *T. tambra*25_F | ACACTCCTCTGCGATTTGGG | 62°C,30 s. | 510 bp. |
|  | *T. tambra*25_R | TTGCAAACGCCACCCAAAAA |  |  |
| 32 | *T. tambra*26_F | ATAGCCCTTCTGCCAGAGGA | 63°C,30 s. | 564 bp. |
|  | *T. tambra*26_R | GAAGAGTCCCTGACACTGGC |  |  |
| 33 | *T. tambra*27_F | AGGCTAGGCTACTGGTGGAA | 63°C,30 s. | 576 bp. |
|  | *T. tambra*27_R | CAGGCTACTGTCTGCTGCAT |  |  |
| 34 | *T. tambra*28_F | TCCCATGACTCGCTTTGACC | 63°C,30 s. | 469 bp |
|  | *T. tambra*28_R | GTCCATCCCTCGACGTGATC |  |  |
| 35 | *T. tambra*29_F | CCTTCTGGGGGCCAAAGTAT | 61°C,30 s. | 570 bp. |
|  | *T. tambra*29_R | GCGATTTGCTGAAATGTGCC |  |  |
| 36 | *T. tambra*30_F | TGCGTAAGTCCTTGTGCTGA | 62°C,30 s. | 564 bp. |
|  | *T. tambra*30_R | GCCTCAGACTGGACCTTTCC |  |  |
| 37 | *T. tambra*31_F | GCAGAGGAGGAGTAAACAACCT | 62°C,30 s. | 537 bp. |
|  | *T. tambra*31_R | GTGGCAGGAGATTCAGCTGT |  |  |
| 38 | *T. tambra*32_F | TGCACGGCATTGCTCCTATT | 65°C,30 s. | 449 bp. |
|  | *T. tambra*32_R | GTTCTAGAGCCACTGACAGCA |  |  |

**Table S2** List of cypriniform genomes used for comparative genomic, phylogenomic, and genome-wide similarity analyses.

| **No.** | **Species** | **Assembly accession** | **Assembly level** | **Analysis use** |
| --- | --- | --- | --- | --- |
| 1 | *Danio rerio* | GCF_049306965.1 | Chromosome-level | Phylogenomics, Mash |
| 2 | *Ctenopharyngodon idella* | GCF_019924925.1 | Chromosome-level | Phylogenomics, Mash |
| 3 | *Hemibarbus labeo* | GCA_051403015.1 | Chromosome-level | Phylogenomics, Mash |
| 4 | *Tor tambroides* | GCA_012432095.1 | Scaffold-level | Phylogenomics, Mash |
| 5 | *Tor tambra* (male; this study) | JARWAD000000000 | Scaffold-level | Phylogenomics, Mash |
| 6 | *Tor tambra* (female; this study) | JARWAE000000000 | Scaffold-level | Mash |
| 7 | *Onychostoma macrolepis* | GCF_012432095.1 | Chromosome-level | Phylogenomics, Mash |
| 8 | *Squaliobarbus curriculus* | GCA_046118805.1 | Chromosome-level | Phylogenomics, Mash |
| 9 | *Ancherythroculter nigrocauda* | GCA_036281575.1 | Scaffold-level | Phylogenomics, Mash |
| 10 | *Xenocypris davidi* | GCA_048182625.1 | Chromosome-level | Phylogenomics, Mash |
| 11 | *Labeo catla* | GCA_012976165.1 | Scaffold-level | Phylogenomics, Mash |
| 12 | *Onychostoma simum* | GCF_000283155.1 | Scaffold-level | Phylogenomics, Mash; rooting reference |

**Table S3. Full BUSCO results for male and female *Tor tambra* genome assemblies using the actinopterygii_odb10 dataset.**

| **Assembly** | **Complete BUSCOs (%)** | **Complete single-copy (%)** | **Complete duplicated (%)** | **Fragmented (%)** | **Missing (%)** | **Total BUSCO groups searched** | **Lineage dataset** |
| --- | --- | --- | --- | --- | --- | --- | --- |
| Male | 96.2 | 56.4 | 39.8 | 1.1 | 2.7 | 3640 | actinopterygii_odb10 |
| Female | 96.2 | 57.5 | 38.7 | 0.9 | 2.9 | 3640 | actinopterygii_odb10 |

**Table S4. Summary of gene prediction and annotation coverage.**

| **Genome** | **Total Genes** | **With COG Annotation** | **With KEGG Pathway** | **With PFAM Domain** |
| --- | --- | --- | --- | --- |
| **Male** | 50,705 | 49,155 (96.9%) | 18,658 (36.8%) | 48,374 (95.4%) |
| **Female** | 49,864 | 48,378 (97.0%) | 18,533 (37.2%) | 47,575 (95.4%) |

**Table S5** Distribution of genes by COG functional category.

| COG Category | Functional Class | Male | Female |
| --- | --- | --- | --- |
| S | Function unknown | 19,756 | 19,113 |
| T | Signal transduction mechanisms | 7,782 | 7,757 |
| K | Transcription | 3,087 | 3,089 |
| O | Posttranslational modification, protein turnover, chaperones | 2,896 | 2,848 |
| L | Replication, recombination and repair | 1,981 | 1,985 |
| G | Carbohydrate transport and metabolism | 1,603 | 1,594 |
| E | Amino acid transport and metabolism | 1,472 | 1,453 |
| P | Inorganic ion transport and metabolism | 1,012 | 995 |
| C | Energy production and conversion | 978 | 972 |
| J | Translation, ribosomal structure, and biogenesis | 858 | 861 |

**Table S6** Summary of SSR identification statistics for male and female *Tor tambra* genomes.

| **Feature** | **Male** | **Female** |
| --- | --- | --- |
| Total SSRs identified | 172,934 | 175,958 |
| Total sequence size (bp) | 1,250,616,913 | 1,244,113,601 |
| SSR-containing sequences | 4,013 | 3,754 |
| Sequences with >1 SSR | 3,859 | 3,663 |
| SSRs in compound formation | 7,377 | 7,494 |
| Mononucleotide SSRs | 132,685 | 136,017 |
| Dinucleotide SSRs | 24,503 | 24,316 |
| Trinucleotide SSRs | 12,689 | 12,568 |
| Tetranucleotide SSRs | 2,981 | 2,969 |
| Pentanucleotide SSRs | 75 | 87 |
| Hexanucleotide SSRs | 1 | 1 |

**Table S7. Summary of transposable element and repeat composition in male and female *Tor tambra* genomes based on de novo RepeatModeler/RepeatMasker annotation**

| **Repeat category** | **Female: number of elements** | **Female: occupied length (bp)** | **Female: genome (%)** | **Male: number of elements** | **Male: occupied length (bp)** | **Male: genome (%)** |
| --- | --- | --- | --- | --- | --- | --- |
| **Bases masked (total)** | — | 583,979,592 | 46.94 | — | 586,673,803 | 46.91 |
| **Retroelements** | 118,475 | 84,410,978 | 6.78 | 119,237 | 84,512,538 | 6.76 |
| SINEs | 5,511 | 1,163,419 | 0.09 | 5,503 | 1,147,700 | 0.09 |
| Penelope | 0 | 0 | 0.00 | 0 | 0 | 0.00 |
| LINEs | 81,394 | 46,540,420 | 3.74 | 81,861 | 46,825,714 | 3.74 |
| CRE/SLACS | 0 | 0 | 0.00 | 0 | 0 | 0.00 |
| L2/CR1/Rex | 63,393 | 37,147,724 | 2.99 | 63,719 | 37,277,668 | 2.98 |
| R1/LOA/Jockey | 2,707 | 1,001,784 | 0.08 | 2,685 | 1,003,115 | 0.08 |
| R2/R4/NeSL | 2,205 | 773,406 | 0.06 | 2,430 | 831,916 | 0.07 |
| RTE/Bov-B | 2,959 | 1,205,196 | 0.10 | 3,020 | 1,215,418 | 0.10 |
| L1/CIN4 | 2,624 | 2,514,718 | 0.20 | 2,634 | 2,580,773 | 0.21 |
| LTR elements | 31,570 | 36,707,139 | 2.95 | 31,873 | 36,539,124 | 2.92 |
| BEL/Pao | 709 | 1,088,687 | 0.09 | 704 | 1,084,462 | 0.09 |
| Ty1/Copia | 0 | 0 | 0.00 | 0 | 0 | 0.00 |
| Gypsy/DIRS1 | 26,207 | 26,844,283 | 2.16 | 26,603 | 27,137,224 | 2.17 |
| Retroviral | 4,419 | 8,518,908 | 0.68 | 4,319 | 8,045,719 | 0.64 |
| **DNA transposons** | 131,353 | 59,465,871 | 4.78 | 131,291 | 59,445,622 | 4.75 |
| hobo-Activator | 8,142 | 1,370,939 | 0.11 | 8,216 | 1,373,002 | 0.11 |
| Tc1-IS630-Pogo | 76,583 | 45,473,328 | 3.66 | 76,482 | 45,710,762 | 3.66 |
| En-Spm | 0 | 0 | 0.00 | 0 | 0 | 0.00 |
| MULE-MuDR | 0 | 0 | 0.00 | 0 | 0 | 0.00 |
| PiggyBac | 2,292 | 414,241 | 0.03 | 2,284 | 407,596 | 0.03 |
| Tourist/Harbinger | 5,943 | 1,555,300 | 0.13 | 6,079 | 1,611,979 | 0.13 |
| Other (Mirage, P-element, Transib) | 180 | 61,811 | 0.00 | 184 | 56,964 | 0.00 |
| Rolling circles | 0 | 0 | 0.00 | 0 | 0 | 0.00 |
| **Unclassified** | 2,066,918 | 404,700,479 | 32.53 | 2,074,134 | 407,026,907 | 32.55 |
| **Total interspersed repeats** | — | 548,577,328 | 44.09 | — | 550,985,067 | 44.06 |
| Small RNA | 25,350 | 4,592,034 | 0.37 | 25,542 | 4,461,910 | 0.36 |
| Satellites | 2 | 2,066 | 0.00 | 1 | 838 | 0.00 |
| Simple repeats | 607,021 | 27,190,427 | 2.19 | 609,481 | 27,543,024 | 2.20 |
| Low complexity | 66,220 | 3,617,737 | 0.29 | 66,930 | 3,682,964 | 0.29 |

**Table S8.** Sensitivity analysis of zero-coverage candidate regions under different mapping-quality thresholds.

| **Sample** | **MAPQ** | **Weighted mean depth (×)** | **Zero-coverage bp** | **% genome** | **Zero-coverage bp ≥500 bp** | **% genome ≥500 bp** |
| --- | --- | --- | --- | --- | --- | --- |
| Male | 10 | 74.31 | 25,424,677 | 2.06 | 12,611,460 | 1.02 |
| Male | 20 | 72.65 | 27,167,715 | 2.20 | 14,271,988 | 1.16 |
| Male | 30 | 70.42 | 29,700,933 | 2.40 | 16,717,024 | 1.35 |
| Female | 10 | 70.91 | 25,487,548 | 2.06 | 12,467,578 | 1.01 |
| Female | 20 | 69.30 | 27,298,331 | 2.21 | 14,172,431 | 1.15 |
| Female | 30 | 67.15 | 29,857,535 | 2.42 | 16,566,458 | 1.34 |

Summary of robustness testing for zero-coverage candidate regions identified by mapping male and female Tor tambra Illumina read sets to the T. tambroides reference genome under three alignment-quality filters (MAPQ ≥10, ≥20, and ≥30). For each condition, weighted mean depth, total zero-coverage sequence, and the proportion of the reference genome represented by zero-coverage intervals were calculated. Results are shown for all zero-coverage intervals and for intervals ≥500 bp to reduce the influence of short stochastic gaps. The analysis demonstrates that the exact estimate is parameter-dependent but remains consistently low across reasonable filtering thresholds.


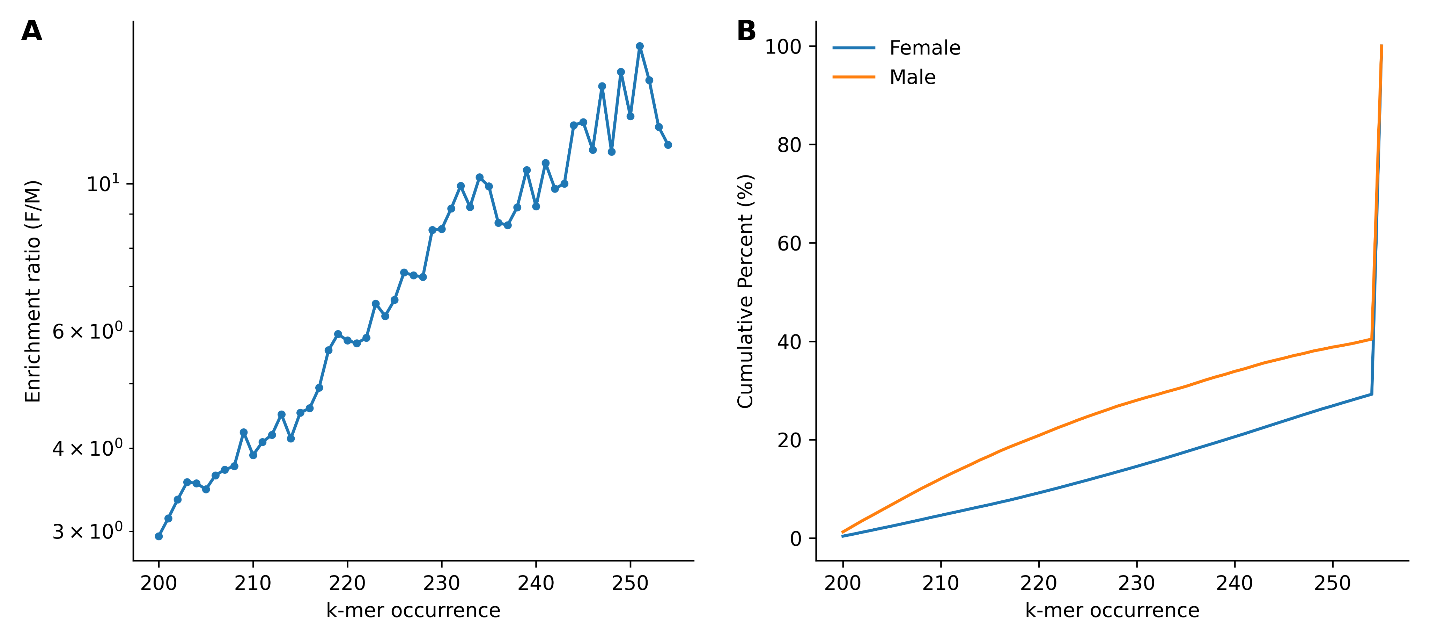


**Figure S1. Female-biased enrichment of moderate-copy sex-specific k-mers.** (A) Enrichment ratio (Female/Male) across k-mer occurrence counts (k = 31; 200–254 occurrences). The y-axis is shown on a log scale. Enrichment increases progressively with copy number, reaching a maximum ratio of 16.1 at high-copy bins, suggesting increased representation of female-associated sequences under the applied filtering criteria. (B) Cumulative distribution (ECDF) of female- and male-specific k-mers across occurrence counts (200–255). The sharp increase at the rightmost bin reflects count saturation at ≥255 (8-bit storage ceiling), where highly repetitive k-mers accumulate. Across most of the moderate-copy range, female-specific k-mers consistently outnumber male-specific k-mers, indicating a detectable female-biased enrichment signal under the applied filtering parameters.


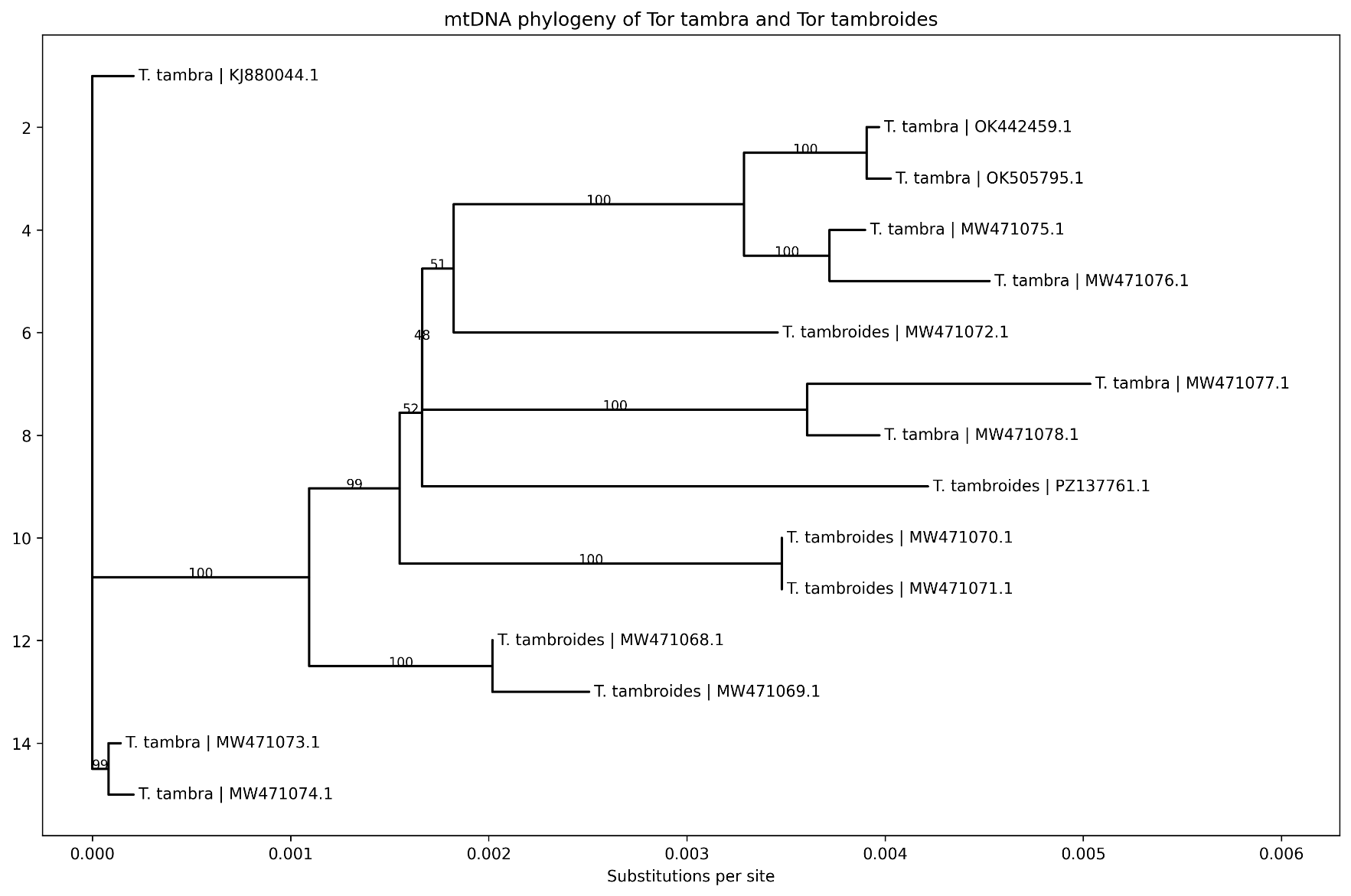


**Figure S2.** Maximum-likelihood phylogeny inferred from 15 complete mitochondrial genomes of *Tor tambra* and *Tor tambroides*. The mtDNA tree did not recover clean reciprocal monophyly of the two nominal species. Although some local lineages formed compact clusters, mitochondrial haplotypes were interspersed between *T. tambra* and *T. tambroides*, indicating limited species-level resolution of mitochondrial genomes in this comparison. This contrasts with the BUSCO-based nuclear phylogeny, which resolved the two taxa as distinct nuclear lineages.

**Supplementary Script S1. MaSuRCA configuration file and execution commands for hybrid assembly of the female *Tor tambra* genome**

# MaSuRCA v4.1.2 hybrid assembly of female Tor tambra genome

**# Step 1: create MaSuRCA configuration file**

cat > masurca_female_config.txt <<'EOF'

# quick run configuration file

DATA

PE = pe 500 50 out.R1.fq.gz out.R2.fq.gz

NANOPORE = female_nanopore.fastq.gz

END

PARAMETERS

EXTEND_JUMP_READS=0

GRAPH_KMER_SIZE=auto

USE_LINKING_MATES=0

USE_GRID=0

GRID_ENGINE=SGE

GRID_QUEUE=all.q

GRID_BATCH_SIZE=500000000

LHE_COVERAGE=25

LIMIT_JUMP_COVERAGE=300

CA_PARAMETERS=cgwErrorRate=0.15

CLOSE_GAPS=1

NUM_THREADS=50

JF_SIZE=200000000

SOAP_ASSEMBLY=0

FLYE_ASSEMBLY=0

END

EOF

**# Step 2: generate assembly workflow**

masurca masurca_female_config.txt

**# Step 3: run assembly**

bash assemble.sh

**Supplementary Script S2. BRAKER2 command used for structural gene prediction**

# BRAKER2 v2.1.6 gene prediction using RNA-seq alignments

# and Cyprinidae protein evidence on the soft-masked female genome

braker.pl \

--genome=Tor_tambra_female_primary.fa.masked \

--bam=female_all_trimmed.bam \

--prot_seq=cyprinidae.clean.nr.fa \

--softmasking \

--gff3 \

--cores=48 \

--GENEMARK_PATH=/home/mecob/Software/miniconda3/envs/braker_env/bin/gmes_linux_64
